# Factors Affecting Physicians’ Credibility on Twitter When Sharing Health Information: Online Experimental Study

**DOI:** 10.2196/34525

**Published:** 2022-06-13

**Authors:** DaJuan Ferrell, Celeste Campos-Castillo

**Affiliations:** 1 Critical Writing Program University of Pennsylvania Philadelphia, PA United States; 2 Department of Sociology University of Wisconsin-Milwaukee Milwaukee, WI United States

**Keywords:** source credibility, user engagement, social media, health communication, misinformation, Twitter

## Abstract

**Background:**

Largely absent from research on how users appraise the credibility of professionals as sources for the information they find on social media is work investigating factors shaping credibility within a specific profession, such as physicians.

**Objective:**

We address debates about how physicians can show their credibility on social media depending on whether they employ a formal or casual appearance in their profile picture. Using prominence-interpretation theory, we posit that formal appearance will affect perceived credibility based on users' social context—specifically, whether they have a regular health care provider.

**Methods:**

For this experiment, we recruited 205 social media users using Amazon Mechanical Turk. We asked participants if they had a regular health care provider and then randomly assigned them to read 1 of 3 Twitter posts that varied only in the profile picture of the physician offering health advice. Next, we tasked participants with assessing the credibility of the physician and their likelihood of engaging with the tweet and the physician on Twitter. We used path analysis to assess whether participants having a regular health care provider impacted how the profile picture affected their ratings of the physician’s credibility and their likelihood to engage with the tweet and physician on Twitter.

**Results:**

We found that the profile picture of a physician posting health advice in either formal or casual attire did not elicit significant differences in credibility, with ratings comparable to those having no profile image. Among participants assigned the formal appearance condition, those with a regular provider rated the physician higher on a credibility than those without, which led to stronger intentions to engage with the tweet and physician.

**Conclusions:**

The findings add to existing research by showing how the social context of information seeking on social media shapes the credibility of a given professional. Practical implications for professionals engaging with the public on social media and combating false information include moving past debates about casual versus formal appearances and toward identifying ways to segment audiences based on factors like their backgrounds (eg, experiences with health care providers).

## Introduction

### Background

Policy makers, journalists, researchers, and industry leaders have promoted social media as a catalyst for revolutionizing health care by extending the reach of health advice from physicians [1-7]. Notwithstanding the persistence of digital divides in who has internet access and uses social media [8,9], the focus appears well-placed given that internet users turn to social media for health advice [4,10,11] and that many report improvements in their health as a result [2-6,12]. However, there is little research to date investigating what impacts physicians’ credibility as a source of health advice on social media, with most research focusing on only comparing the credibility of physicians versus other sources [10]. The credibility of a source refers to the degree to which the information it supplies is believable [13]. Understanding variations in credibility among physicians on social media is important given the need to combat misinformation there. This is particularly important during public health emergencies like the COVID-19 pandemic [14].

Best practices for how professionals can cue their credibility on social media are unclear [15-18]. This is because on social media platforms for health advice like Twitter, the norm is to present yourself as an approachable peer, while in professional settings, the norm is distinguishing yourself from the lay public to signify you are an authority. For physicians, the competing norms fuel philosophical debates over how to leverage social media to strengthen their connections with the public while also presenting themselves in a way that adheres to medical ethics [19-21]. As a practical matter, the presentation norms prescribe contrasting strategies for populating one’s own social media account, like whether one should post casual or formal pictures of themselves [19-22]. No study to date has compared how the 2 strategies shape the credibility of a physician sharing health advice on social media.

Thus, we conducted an experiment addressing how a casual and formal appearance may shape a physician’s credibility on Twitter when sharing health advice through a tweet. We investigated the complexity in this process by examining how the importance of a casual and formal appearance for physician credibility depends on whether a user has a regular health care provider. Moreover, we examined how the effects of appearance on credibility in turn affect the likelihood that a user engages with the tweet. Findings contribute to theorizing how social context influences credibility judgments during information seeking through amplifying cues (eg, formal appearance), as well as discussions about online presentation strategies and ways physicians can aid in inoculating against falsehoods (ie, misinformation and disinformation) on social media. Moreover, as health professionals turn to the internet to provide care during the COVID-19 pandemic [23], knowing the factors that signal a physician’s credibility and inoculating against falsehoods on online media have become more critical.

### Prior Work

The social media ecosystem creates a decentralized information environment where access to information is mediated by nontraditional authorities (eg, friends, family, influential social media users), which consequently spurs questions about source credibility. Social media users are tasked with determining source credibility, which raises concerns that they may engage with or spread false information. Thus, there is a need to research how social media users determine source credibility and how legitimate sources like physicians can leverage findings to share validated information.

#### Credibility on Social Media

To form impressions about the veracity of information shared by a source, individuals use 3 features of the source to determine its credibility [13]: competence, trustworthiness, and goodwill. Competence refers to the source’s ability or qualifications to know the truth regarding a matter. The source’s trustworthiness represents the motivation to be truthful or biased on a matter. Goodwill is the extent that the source has the individual’s best interest at heart.

On social media, users can glean these 3 features constituting credibility by looking for authority cues. The presence of credentials, such as a badge, organizational affiliation and other external links, or a professional title, on a social media profile acts as an authority cue that users rely on to determine whether the source is credible [10,24]. For example, one study found that a Twitter profile sharing information about gonorrhea with cues connecting it to the Center for Disease Control and Prevention resulted in stronger perceptions of competence, trustworthiness, and goodwill than when the profile contained cues signaling the information came from a peer or stranger [25].

#### Physician Credibility

Few studies have examined variation in credibility ratings on social media within a single type of authority operating as a source. For physicians, researchers have studied how 2 different presentation styles—casual versus formal attire—cue their credibility within in-person settings. Patients generally prefer physicians to wear formal attire, like a white coat, rather than casual attire during clinic visits [26], but attire has no significant effect on the credibility ratings of a physician’s treatment recommendations [27].

Although a casual or formal appearance may not matter for cueing physician credibility within in-person settings, the issue becomes more complex within social media and spurs deliberation. The American Medical Association advises physicians to separate their “personal and professional content online” [20]. Profile images where the physician is wearing formal dress (eg, white lab coat, stethoscope) is one way to achieve this since professional symbols like these that indicate the profession [28] crystallize the separation between the public and a profession [29].

Pragmatically, however, this is difficult. This is because the strategies for appearance on social media and a profession can clash, which can diminish an authority’s credibility on social media [16,18,22]. Thus, identifying which strategy is best for cueing a physician’s credibility on social media is critical to improving the reach of factual health advice and inoculating against falsehoods. 

### Conceptual Framework and Hypotheses

#### Hypothesis 1: When Reading a Tweet Sharing Health Advice, Credibility Ratings (Competence, Trustworthiness, and Goodwill) Will Be Higher for Physicians Dressed in Formal Wear Than for Those in Casual Wear Within Their Profile Picture

**Figure 1:**
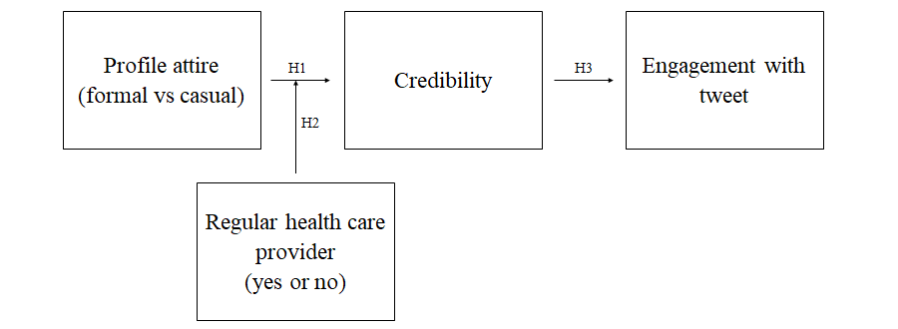
Summary of hypotheses. H: hypothesis.

Figure 1 summarizes our hypotheses. D’Angelo and Van Der Heide [22] found that participants rated a profile from a physician more favorably when the physician was wearing a white lab coat and a stethoscope than when the physician dressed casually. The significant differences in favorability held regardless of whether the profile was on Facebook or a platform with more formal presentation norms, like WebMD (based on our own analysis of their descriptives). They did not examine how a casual and formal appearance shaped credibility ratings in the context of physicians offering health advice on social media, which we investigate in this study. Because studies examining other professionals besides physicians show a formal appearance on social media promotes higher credibility ratings than does a casual appearance [16,30,31], there is a possibility that the findings from D’Angelo and Van Der Heide [22] extend to other contexts.

#### Hypothesis 2: When Reading a Tweet Sharing Health Advice From a Physician Dressed in Formal Wear Within Their Profile Picture, Credibility Ratings (Competence, Trustworthiness, and Goodwill) Will Be Higher Among Users With a Regular Provider Than Among Those Without One

An approach missing from debates about whether physicians should use a formal appearance on social media is to consider when formal appearance may be effective. Prominence-interpretation theory [32] claims users’ past experiences can shape their interpretation of cues online and, in turn, their credibility ratings. For social media users with previous exposure to symbols that are emblematic of physicians, formal wear may be more critical for credibility.

Indirect support for this expectation comes from research on patient preferences for physician attire during a clinic visit. A systematic review of studies sampling patients found that patients express fewer preferences when asked following a clinical visit versus when asked only to imagine experiencing one while they sat in a clinical setting [26]. Patients in the latter case were more likely to prefer formal attire, such as a white lab coat. This is indirect support because in both cases, patients would have been exposed to symbols emblematic of physicians (either during the clinic visit or while waiting). Among these patients, only those asked to imagine an interaction with physicians—akin to the imagined or parasocial interaction that occurs within technology-mediated communication [33,34]—tended to prefer formal attire.

To distinguish between individuals with and without exposure to professional symbols, we asked whether they have a regular health care provider, defined as a physician or other health care professional (eg, nurse, nurse practitioner, physician assistant). The regular provider need not be a physician for this process because the lay public often refers to their regular provider as “doctor” regardless of the type of provider [35,36]. Since professional symbols representing physicians (eg, white coat) are likely more accessible among those with a regular provider than those without, we expected formal attire would be more important for cueing credibility among those with a regular provider than among those without one.

#### Hypothesis 3: As Ratings of Physician Credibility (Competence, Trustworthiness, and Goodwill) Increase, Intentions To Engage With The Physician on Twitter and the Tweet Sharing Health Advice Will Strengthen

Last, we examined how credibility ratings of a physician sharing health advice through a tweet would influence the intentions to engage with the tweet. As Figure 1 shows, the direct predictor of engagement within this model is level of credibility. Because we outlined how appearance in a profile picture and having a regular provider affect credibility, these 2 operate as indirect predictors of engagement. Credible sources are more likely to persuade others by changing their attitudes and behavior [37], with prior research showing the credibility of a source on social media to be associated with the strength of the resulting attitudes and behavioral intentions [38-41].

## Methods

### Ethics Approval

This study was approved by the University of Wisconsin-Milwaukee’s Institutional Review Board (review number 18.045).

### Experimental Design

We conducted a 2 (has regular provider: yes vs no) × 3 (profile attire: no profile image vs casual vs formal) between-subject online experiment to test our hypotheses. Along with enquiring about other demographic variables, we asked participants to report whether they had a regular provider and then randomly assigned them to read 1 of 3 tweets varying only the attire of the physician displayed in the profile image. We then asked them to assess the credibility of the physician and the likelihood that they would engage with the tweet and the physician on Twitter.

### Recruitment

On October 9 and 10 in 2018, we recruited social media users living in the USA from Amazon Mechanical Turk (MTurk) with at least a 95% approval rate. Compared to other convenience samples (eg, college students) used in research to study source credibility and health behaviors, respondents from MTurk tend to be more demographically diverse [42]. Notably, they are more likely to read instructions more carefully than are other convenience samples [43] and therefore may be more attuned to differences in presentation styles. Respondents from MTurk are also more likely to report poorer health overall [44], suggesting they may be more in need of accessing health advice.

We analyzed data from the 205 respondents who passed a series of questions designed to assess the quality of their responses. A power analysis using G*Power software showed this sample size would provide enough power (>0.80) to detect an effect size (0.40) on par with studies comparing the credibility of physicians versus peers on social media [10] with an α level of .05. Of the 205 respondents, approximately 78.% (160/205) identified as White, 7.3% (15/205) as Black, (16/205) 7.8% as Latino, and 6.8% (14/205) as another race or ethnicity. Approximately 43.9% (90/205) reported they were female. The mean age was 36, with the youngest participant reporting an age of 20 years and the oldest reporting an age of 69 years. When asked to rate their overall physical health on a 5-point Likert scale (1=poor, 5=excellent) [45], most (40%, 82/205) selected the “good” option.

### Profile Attire

**Figure 2:**
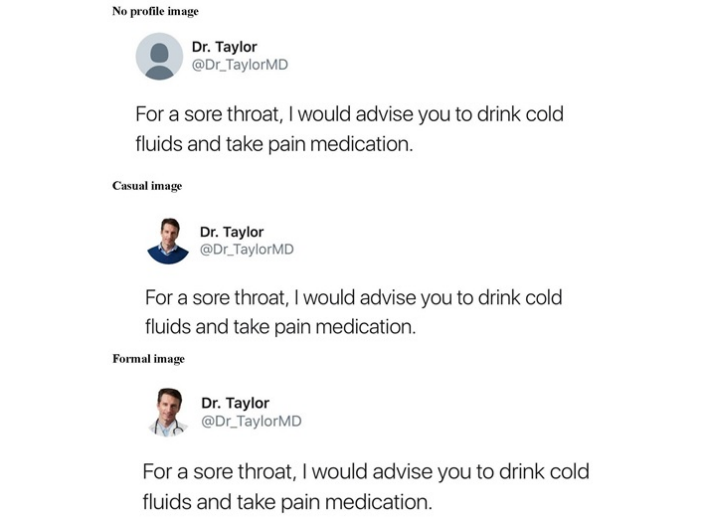
Profile attire conditions.

Figure 2 shows the 3 tweets used in this study, each of which refer to the physician as “Dr.” We designed the content of the tweets that remained consistent across conditions to represent what the average user on Twitter is likely to see. For the tweet, we created a text post that stated, “For a sore throat, I would advise you drink cold fluids and take pain medication.” The text of the post is based on previous research [46] and shares health advice regarding a sore throat, which is a common symptom people experience [47]. Thus, we designed the tweet to present empirically supported information. We selected a male physician, as female physicians are more likely to use Twitter to network with same-gender colleagues and mentors as opposed to sharing medical advice because they are motivated to use the platform to improve their mobility within the profession [48,49]. Moreover, we chose a picture of a physician who appeared to be White and under the age of 55 as most professionals in this field fall within this racial category and age range in the United States [50,51].

The formal and casual conditions used an image of the same male, which we manipulated to alter only his attire and thereby control for confounding variables like attractiveness. In the casual condition, he wore a blue sweater and collared shirt without a tie, while in the formal condition, he wore a white lab coat and a stethoscope, which are symbols associated with the medical professional [28].

Our design incorporated a third condition, the no-profile condition, which contained no profile image of the physician. If there was a nonsignificant difference in the effects of the casual and formal conditions on credibility ratings, the no-profile condition would offer a useful baseline to guide interpretation. A nonsignificant difference would suggest the dress styles exert comparable effects, but perhaps also that the styles do not add significantly to the cues conveying who the source is (eg, the “Dr.” title), which could also affect credibility ratings. Because of the widespread diffusion of medical symbols through media channels [52], it may be that only the cues conveying who the source is are necessary to establishing credibility, and thus images conveying dress style are redundant and exert negligible effects. Such information would also be useful for quelling debates about physicians’ self-presentation on social media by indicating that the 2 styles in practice produce comparable credibility ratings.

### Regular Provider

We used a question that is commonly used in self-reports to identify whether participants had a regular provider [53]: “Not including psychiatrists and other mental health professionals, is there a particular doctor, nurse, or other health professional you see most often?” The measure is dichotomous (1=yes, 0=no).

### Measures

#### Credibility Ratings of Physician

A scale from McCroskey and Teven [13] measures the 3 features that compose a source’s credibility: competence, goodwill, and trustworthiness. Each feature is measured with six 7-point semantic differential questions, totaling 18 survey items.

#### Engagement

Using 7-point Likert questions, we asked participants their intentions to engage with the tweet [54,55], specifically asking how likely they would be to like the tweet, retweet the tweet, share the tweet, and follow the physician.

### Statistical Analysis

The analysis was conducted using Stata 16 (StataCorp). We began with a preliminary analysis, which readers can find in Multimedia Appendix 1. For the preliminary analysis, we conducted randomization checks to ensure that the number of participants with and without a regular provider was neither associated with demographics nor the profile attire conditions. We ended this phase by conducting exploratory factor analyses of the credibility and engagement items to assess their factor structure and calculate factor scores because how people construct credibility can vary [56] and thereby produce changes in factor structure based on situational context [22]. For the main analysis, summarized here in the main text, we estimated 2 path models to test our hypotheses, one with an interaction between profile attire and having a regular provider (testing hypothesis 2) and another without the interaction (testing hypothesis 1 and hypothesis 3). We estimated the path models using the “sem” command and 5000 bootstrap samples. Statistical significance is based on 2-tailed tests and an α level set at .05.

## Results

### Hypothesis Testing

The estimates for the first path model testing hypotheses are depicted in Table 1. The path estimates for the casual and formal attire conditions summarize the predicted levels of the 2 credibility factors for respondents in these conditions relative to levels for respondents in the no-profile condition. Thus, to assess whether credibility ratings were higher for physicians with a formal appearance than those with a casual appearance and thereby test hypothesis 1, a Wald test was used to determine whether the path estimate for the formal condition was significantly greater in magnitude than was the corresponding one for the casual condition. For neither the goodwill (*χ*^2^_1_=0.18, N=205; *P*=.67) nor the competence or trustworthiness factor scores (χ^2^_1_=1.95; N=205; *P*=.16), were the path estimates significantly different. A 1-way multivariate analysis of variances comparing the means for the 2 credibility factor scores between participants in the casual and formal conditions produced the same conclusion (*F*_2_=1.09, N=132; *P*=.34; Wilks' Λ=0.984). The conclusions did not change when we removed the paths estimating the relationships between having a regular provider and the 2 credibility factor scores. These findings fail to support hypothesis 1, suggesting that neither type of appearance is more effective than the other for cueing the credibility of physicians on Twitter.

**Table 1:** Path model estimating the effects of profile attire on engagement without conditional effects of having a regular provider.

Path	Coefficient, *b*	*z* score	*P* value^a^
Casual → competence/trustworthiness	0.239	1.40	.16
Formal → competence/trustworthiness	0.001	0.01	.99
Regular provider → competence/trustworthiness	0.139	1.00	.32
Casual → goodwill	–0.154	–0.91	.37
Formal → goodwill	–0.226	–1.35	.18
Regular provider → goodwill	0.159	1.15	.25
Competence /trustworthiness → engagement	0.030	0.38	.71
Goodwill → engagement	0.382	4.80	<.001

^a^Two-tailed test.

A comparison of the credibility ratings in these conditions to those in the no-profile condition sheds additional insight into this finding. Table 1 shows that compared to participants in the no-profile condition, those in the casual (b=0.239; *P*=.16) and formal (b=0.001; *P*=.99) conditions did not rate the physician’s competence or trustworthiness differently. A similar pattern emerged for ratings of the physician’s goodwill (casual: b=–0.154 and *P*=.36; formal: b=–0.226, *P*=.18). Thus, not only is neither type of appearance more effective than the other for cueing the credibility of physicians on Twitter, but also neither style adds significantly to the baseline credibility established from a source with “Dr.” in the title.

Table 1 also shows results from a test of hypothesis 3, which is that credibility ratings will be positively associated with intentions to engage with the tweet. Only goodwill ratings had a significant association, with higher ratings associated with stronger intentions (b=0.382; *P*<.001). Since the goodwill and competence or trustworthiness factors are strongly correlated, we assessed multicollinearity in the equation-predicting engagement. Multicollinearity could explain why only the goodwill factor was significantly associated with engagement. We calculated the variance inflation factor for both, and each was below the 2.50 recommended threshold [57]. This suggests multicollinearity in the engagement equation is not an issue and that only goodwill is significantly associated with engagement. The findings partially support hypothesis 3.

**Figure 3:**
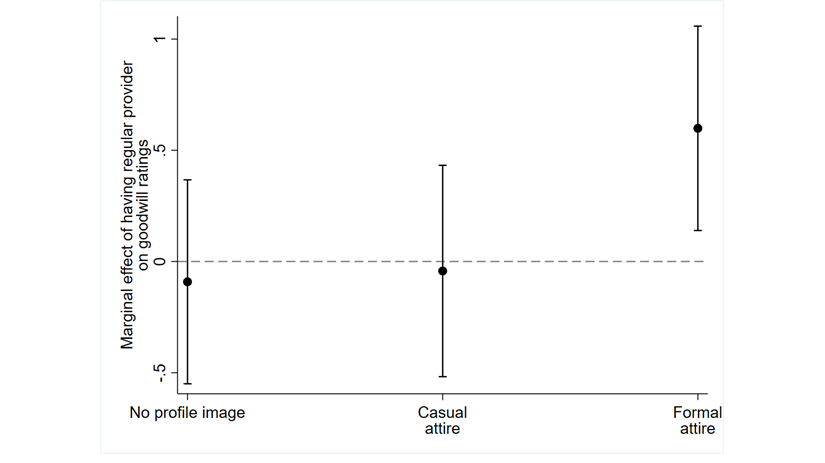
The marginal effect of having a regular provider by profile attire condition, with 95% CIs.

To test hypothesis 2, we estimated a second path model, summarized in Table 2. Hypothesis 2 states that the relative effectiveness of a formal appearance on credibility ratings is contingent on users’ experience with professional symbols, which we operationalized as whether they have a regular provider. Table 2 shows a significant interaction in the equation estimating goodwill ratings between the formal condition and having a regular provider (b=0.690; *P*<.05). To better understand this finding, Figure 3 plots the marginal effect of having a regular provider on predicted goodwill ratings by condition, with 95% CIs. A marginal effect crossing the zero threshold (denoted by a horizontal dashed line) indicates no significant difference in goodwill ratings between those with and without a regular provider. Marginal effects above the threshold represent significantly higher goodwill ratings for those with a regular provider relative to those without, while marginal effects below the threshold represent significantly lower ratings. Figure 3 shows that among participants in the formal condition, those reporting they had a regular provider had significantly higher goodwill ratings than those who said they did not have a regular provider. Goodwill ratings did not significantly differ by whether participants had a regular provider in the other 2 conditions. The results support hypothesis 2 and indicate formal attire in a Twitter profile picture can cue credibility for physicians among users with a regular provider.

Given that earlier we showed goodwill ratings were positively associated with intention to engage with the tweet, we explored indirect effects to assess whether this significant interaction translated into measurable differences in intentions to engage with the tweet between participants with and without a regular provider. Among participants in the formal condition, those with a regular provider had significantly stronger intentions to engage with the tweet than did those who did not (effect 0.265; SE 0.119; 95% CI 0.032-0.499).

**Table 2:** The estimates for the path model summarizing effects of profile attire on engagement with conditional effects of having a regular provider.

Path	Coefficient, *b*	*z* score	*P* value^a^
Casual → competence/trustworthiness	0.194	0.85	.40
Formal → competence/trustworthiness	–0.032	–0.13	.89
Regular provider × casual → competence/ trustworthiness	0.086	0.28	.78
Regular provider × formal → competence/ trustworthiness	0.063	0.18	.86
Regular provider → competence/trustworthiness	0.090	0.33	.74
Casual → goodwill	–0.184	–0.69	.49
Formal → goodwill	–0.597	–2.45	.01^b^
Regular provider × casual → goodwill	0.049	0.16	.88
Regular provider × formal → goodwill	0.690	2.48	.01^b^
Regular provider → goodwill	–0.091	–0.42	.68
Competence/trustworthiness → engagement	0.030	0.46	.65
Goodwill → engagement	0.382	5.02	<.001^b^

^a^Two-tailed test.

^b^*P*<.05.

## Discussion

### Principal Findings

The growing concern over misinformation and disinformation regarding health information [58,59] on social media raises the need to understand the factors that cue the credibility of authorities. For authorities like physicians and other professionals, a formal appearance potentially clashes with the casual norms on social media platforms like Twitter and thereby risks lowering their credibility. Alternatively, a formal appearance can bolster credibility, helping users evaluate the veracity of the information shared by the physician.

In our study, findings from the experiment varying whether a physician sharing health advice on Twitter wore formal or casual attire in a profile image showed no significant differences in credibility ratings, and, further, these ratings were not significantly different from a condition without a profile image. However, among participants who were shown a physician with formal attire, those reporting that they have a regular provider gave the physician higher credibility ratings than did individuals without one, which in turn led to stronger intentions to engage with the tweet. This pathway operated through a specific credibility rating, goodwill, indicating the importance of this credibility factor for engaging with health advice on social media. The findings are important for advancing theories of source credibility on social media and practitioners interested in combating false information, both of which are critical endeavors to curb the spread of widespread disease (eg, during the global COVID-19 pandemic).

On average, whether a physician used a formal appearance displaying symbols in their profile picture that are emblematic of their profession (eg, white lab coat, stethoscope) had little bearing on their credibility ratings, with ratings comparable to a physician with no profile picture. A physician with a casual appearance likewise had similar credibility ratings as one with no profile picture. In all conditions, the physician was labeled “Dr.,” indicating the contribution of visual symbols in a profile picture did not significantly add to the credibility stemming solely from the physician’s title. The finding implies that debates about how physicians should present themselves on Twitter [19-21] have little practical relevance, at least with respect to decisions about one’s profile image.

We found a key qualifier to the effects of appearance, whereby having a regular provider amplified the effect of a formal appearance on a physician’s credibility ratings, specifically leading to higher goodwill scores compared to those without a regular provider. This finding aligns with prominence-interpretation theory [32] by showing how users’ experiences modulate the relationship between cues and credibility. The result is a demonstration of how context alters the meaning assigned to cues, which in turn results in disparate credibility judgments of the same professional. We therefore echo others in recommending efforts to segment users based on their backgrounds to promote engagement with social media content [7].

The conditional effects we found for formal appearance produced significant differences in intentions to engage with the tweet and the physician posting it. Specifically, a formal appearance shaped intentions to engage among participants with a regular provider through altering ratings of only the goodwill and not the combined competence-trustworthiness factor. Like another study analyzing impressions of physicians on social media [22], an exploratory factor analysis of the items measuring credibility ratings suggested that the items represented 2 instead of 3 factors. However, whereas this other study found that the trustworthy items aligned with the goodwill items, we found they aligned with the competence items. The different factor structure may be because of the different social media platforms under investigation (Twitter vs Facebook and WebMD), samples (MTurk workers vs college students), or gender of the physician (male vs female) but could also be attributable to the different task contexts [32]. The context for our study was to decide whether to engage with a tweet sharing health advice, which may strengthen the link between perceptions of competence and trustworthiness. Conversely, participants in the other study were only asked to judge the profiles of physicians, which might not have associated competence and trustworthiness to the same degree. In line with this interpretation is research from 2 different lines. The first shows people can vary in how they construct credibility depending on context [56]. The second shows the extent that a person’s perceived competence and a term related to goodwill—benevolence—inform perceptions of the individual’s trustworthiness and is also contingent on context [60].

### Limitations and Future Research

We were unable to evaluate how the gender of the physician may moderate findings because we only examined tweets by a male physician. Previous studies reported few differences by physicians’ gender in patient preferences for attire [26]. One study [22] found that a female physician wearing a white lab coat with a stethoscope on her Facebook profile image received higher favorability ratings (a measure that included credibility ratings, among other ratings) than did one wearing a short-sleeved casual shirt. This aligns with our finding showing credibility ratings were higher in the formal than in the casual attire condition. We therefore suspect the conditional importance of formal attire we found will be comparable for tweets by a female physician, but future research should conduct a direct test. Moreover, additional factors, like the perceived age and race of the physician, may likewise shape findings, which future research may examine.

### Conclusions

Although this study was conducted before the discovery and spread of COVID-19 across the globe, the findings are still relevant. Many people turned to the internet to learn information about the virus and government responses [61,62]. Social distancing and stay-at-home orders to curb the spread of the virus led to a dramatic drop in in-person clinic visits [63]. These changes amplified the need to understand how best to disseminate health advice over the internet. Our findings suggest that, on average, a formal and casual appearance influence physician credibility comparably. However, for those with a regular provider, formal dress can raise physician credibility. Indeed, during the rapid uptake of telehealth during the pandemic [63], patients were asking their physicians whether they were wearing their white lab coat [64]. After COVID-19 is controlled, the need to understand how best to support communication between patients and their providers over the internet will remain, along with the need to combat false information about diseases and mitigation strategies. Segmentation strategies [7] will also be key because users’ backgrounds provide relevant contexts shaping how they interpret cues and engage with content. By understanding the factors influencing credibility within a specific authority, this study is one critical step toward those efforts.

## Appendix

Multimedia Appendix 1 Supplementary material.

## Abbreviations

MTurk: Amazon Mechanical Turk
