# Supplementary material for: Factors Affecting Physicians’ Credibility on Twitter When Sharing Health Information: Online Experimental Study
Source: JMIR Infodemiology. 2022 Jun 13;2(1):e34525. doi: 10.2196/34525 (PMC9987183; doi:10.2196/34525)
Supplement: Multimedia Appendix 1 [file infodemiology_v2i1e34525_app1.docx]

**Preliminary Analysis**

*Randomization Checks*

Approximately 53% of the sample reported having a regular provider. To evaluate whether sample characteristics were associated with reports of having a regular provider, we conducted an independent sample t-test (for age) and chi-square tests of independence (for gender, race/ethnicity, and self-rated health). One participant had a missing value on age and another on race/ethnicity. The t-test showed participant age was not significantly associated with reports of having a regular provider (having a regular provider M = 37.1, SD = 10.3; not having a regular provider M = 35.7, SD = 9.8), *t*(202) = 0.982, *p* = 0.33. The chi-square tests showed participant gender, χ^2^ (1, N = 205) = 0.200, *p* = 0.655, race/ethnicity, χ^2^ (4, N = 204) = 2.419, *p* = 0.659, and self-rated health, χ^2^ (4, N = 205) = 6.272, *p* = 0.180, were not significantly associated with reports of having a regular provider.

We also examined whether reports of having a regular provider were associated with assigned study condition. A chi-square test of the independence between participants having a regular provider and being assigned to a profile attire condition was non-significant, χ^2^ (2, N = 205) = 0.169, *p* = 0.919. Altogether, these findings indicate that the number of participants with and without a regular provider was randomly distributed across the three profile attire conditions and that having a regular provider is not associated with sample characteristics.

*Exploratory Factor Analysis*

To examine the factor structure of the 18 items measuring credibility ratings, we conducted a principal component analysis with promax rotation. The analysis suggested a two-factor solution, with eigenvalues for each above 1.5. We retained 13 items with factor loadings above 0.60, summarized in Table 1. The resulting two factors were goodwill and a combined competence/trustworthiness factor that accounted for 82.09% of the variance.

We conducted a principal component analysis with varimax rotation on the four engagement items, summarized in Table 2, which exhibited means comparable to other studies (J. Y. Lee & Sundar, 2013). The eigenvalue for the one factor solution suggested was above 3.0 and all factor loadings were above 0.60. The resulting engagement factor accounted for 83.84% of the variance.

We calculated factor scores for the goodwill, competence/trustworthiness, and engagement factors. The correlations among these three factor scores are summarized in Table 3 and their means and standard deviations across conditions are in Table 4. Given the strong correlation (ρ = 0.59, *p* < .001) between the goodwill and competence/trustworthiness factor scores, we assessed multicollinearity in our path model estimates during hypothesis testing.

| **Table 1.** The means (M), standard deviations (SD), and results from the principal component analysis with promax rotation for retained items measuring credibility. | | | | |
| --- | --- | --- | --- | --- |
|  | M | SD | Competence/Trustworthiness | Goodwill |
| “Please indicate your impression of the physician. The closer the number is to an adjective, the more certain you are of your evaluation.”  Unintelligent-Intelligent | 4.41 | 1.49 | **.91** | -.00 |
| Untrained-Trained | 4.38 | 1.73 | **.99** | -.08 |
| Inexpert-Expert | 4.35 | 1.62 | **.97** | -.07 |
| Uninformed-Informed | 4.44 | 1.70 | **.97** | -.04 |
| Incompetent-Competent | 4.47 | 1.66 | **.92** | .02 |
| Stupid-Bright | 4.52 | 1.52 | **.89** | .05 |
| Doesn’t care about me-Cares about me | 4.35 | 1.52 | .00 | **.95** |
| Doesn’t have my interests at heart-Has my interests at heart | 3.82 | 1.62 | .07 | **.88** |
| Self-centered-Not Self-centered | 4.06 | 1.38 | .18 | **.60** |
| Unconcerned with me-Concerned with me | 3.66 | 1.56 | -.10 | **.99** |
| Dishonest-Honest | 4.46 | 1.48 | **.70** | .27 |
| Untrustworthy-Trustworthy | 4.17 | 1.66 | **.75** | .21 |
| Dishonorable-Honorable | 4.44 | 1.39 | **.70** | .24 |
|  |  |  |  |  |

| **Table 2.** The means (M), standard deviations (SD), and results from principal component analysis with varimax rotation for items measuring engagement with a tweet. | | | | |
| --- | --- | --- | --- | --- |
|  | M | SD | Engagement |  |
| Like it | 2.16 | 1.68 | .91 |  |
| Retweet it | 1.83 | 1.41 | .93 |  |
| Follow | 2.26 | 1.65 | .90 |  |
| Share | 1.89 | 1.38 | .92 |  |
|  |  |  |  |  |

| **Table 3.** The correlations between factor scores. | | | |
| --- | --- | --- | --- |
|  | 1 | 2 | 3 |
| 1. Competence/Trustworthiness | 1.00 |  |  |
| 2. Goodwill | 0.59 | 1.00 |  |
| 3. Engagement | 0.26 | 0.40 | 1.00 |
| Note: All correlations are significant at *p* < .001. | | | |
|  |  |  |  |

| **Table 4.** The means (M) and standard deviations (SD) for factor scores across conditions. | | | | | | | | | | | | | |
| --- | --- | --- | --- | --- | --- | --- | --- | --- | --- | --- | --- | --- | --- |
|  | No provider | | | | | | Provider | | | | | | |
|  | No Profile  N=33 | | Casual  N=32 | | Formal  N=32 | | No Profile  N=37 | | Casual  N=33 | | Formal  N=38 | | |
|  | M | SD | M | SD | M | SD | M | SD | M | SD | M | SD |  |
| Competence  /Trustworthiness | -.12 | 1.10 | .07 | .91 | -.16 | 1.01 | -.03 | .95 | .25 | 1.00 | -.00 | 1.02 |  |
| Goodwill | .17 | 1.16 | -.01 | .92 | -.42 | .99 | .08 | .91 | -.05 | 1.00 | .18 | .95 |  |
| Engagement | .06 | 1.08 | -.07 | .99 | -.16 | .82 | .11 | 1.13 | -.06 | .84 | .08 | 1.10 |  |
|  |  |  |  |  |  |  |  |  |  |  |  |  |  |
